# Measuring access to essential medicines in the sustainable development goals 

**DOI:** 10.2471/BLT.24.291399

**Published:** 2024-08-01

**Authors:** Kristina Jenei, Veronika J Wirtz

**Affiliations:** a Department of Health Policy, London School of Economics and Political Science, Houghton Street, London WC2A 2AE, England.; b World Health Organization Collaborating Center in Pharmaceutical Policy, Boston University School of Public Health, Boston, United States of America.

The World Bank and World Health Organization (WHO) estimate that medicines account for up to 70% of out-of-pocket spending,^1^ and such spending is a major driver of financial hardship.^1^^,^^2^ Despite the barrier medicines pose to achieving universal health coverage (UHC), data to monitor the global situation are lacking. The United Nations (UN) Inter-Agency and Expert Group on sustainable development goal (SDG) indicators started a comprehensive review of the indicators ahead of the 56th United Nations Statistical Commission in 2025. The group announced that indicators with less than 30% data coverage may be subject to deletion, placing the indicator measuring access to medicines at risk of being removed. We call for a renewed commitment to ensure that measuring medicines access does not disappear from the global agenda. 

In 2019, the UN adopted indicator 3.b.3 to evaluate access to medicines by measuring the proportion of health facilities with a core set of relevant essential medicines available and affordable on a sustainable basis.^3^ Currently, the 3.b.3 indicator has the fewest country contributions among the 28 SDG 3 indicators. The WHO database only has 24 country surveys, 23 of which are outdated.^3^ Poor data collection has already affected an indicator measuring access to medicines in the millennium development goals (MDG). Target 8.E only included 26 surveys until 2015,^4^ resulting in its exclusion from six years of MDG reports. Similarly, the lack of data for SDG indicator 3.b.3 has led to its exclusion from the UN Global Sustainable Development Reports,^5^ UHC service coverage index^6^ and academic efforts measuring progress towards UHC.^7^

We offer three reasons for these data gaps. First, the increased number of SDG targets to report may have overburdened countries.^8^ The MDGs included 21 targets and 60 indicators, whereas the SDGs ballooned to 169 targets and 232 indicators. The continued lack of government and donor resources, technical capacity and governance that affected the MDGs also affects the SDGs.^9^

Second, the SDG indicator for access to medicines became more complex in terms of number of items included as well as methods used compared to its predecessor indicator in the MDGs. For instance, a weight for disease burden was added to the SDG indicator numerator to capture demand for medicines as part of availability. However, the weight offers limited value, as availability in the indicator is a binary variable which does not capture volumes needed to match demand over time.^10^ Additional survey instruments, such as the WHO Essential Medicines and Health Products Price and Availability Monitoring Mobile Application (WHO EMP MedMon), were introduced that increase data requirements.

Third, while over the past two decades access to medicines metrics for disease-specific, vertical, donor-supported programmes such as human immunodeficiency virus, tuberculosis and malaria have received much global attention due to their political economy, the same does not apply to access policies and programmes for domestically funded medicines.

Keeping indicator 3.b.3 on the global health agenda is crucial as it is the only metric measuring access to medicines. Indicators with low data availability may need the most investments. The SDG indicators 3.8.1 and 3.8.2 on UHC remain difficult to interpret without measuring the core access dimensions of availability and affordability at facility level via indicator 3.b.3. Low medicines availability at facility level would result in a low UHC index, while low affordability could be the root cause of high financial expenditure.

The 2025 Inter-Agency Expert Group on SDG indicators review is an opportunity to improve data infrastructure and measurement to increase feasibility. The basket of 30 essential medicines chosen in the early 2000s should be reviewed to ensure relevance for priority health issues, for example by adding children’s medicines.^11^ Dropping weight for disease burden should be considered given the aforementioned limitations.^10^

Nationally, countries could build on existing infrastructure such as routine electronic data collection at retail pharmacies. Internationally, WHO could build on the movement for price transparency and the requirements to measure progress towards UHC. The World Health Assembly resolution on *Improving the transparency of markets for medicines, vaccines, and other health products* (WHA 72.8) represents a commitment to share information on the sales, prices, approval status, and research and development of medicines.^12^

Recognizing medicines as a major driver of catastrophic health expenditure requires action on measuring progress towards the SDGs. A policy window has opened with the 2025 Inter-Agency Expert Group on SDGs indicators review that must be used to prevent this indicator from disappearing. 
